# Effects of different harvesting seasons on the physical, chemical, and aromatic properties of tea leaves

**DOI:** 10.1016/j.fochx.2026.104145

**Published:** 2026-06-28

**Authors:** Hicran Uzun Karka, Songul Kesen

**Affiliations:** Gaziantep University, Naci Topçuoğlu Vocational School, 27310 Gaziantep, Turkey

**Keywords:** *Camellia sinensis*, Harvest time, Mineral composition, Aroma compounds, Antioxidant activity

## Abstract

This study evaluated the influence of seasonal harvesting on the physical, chemical, and bioactive characteristics of tea leaves. Moisture content declined from 75.3% in May to 70.5% in September, indicating reduced freshness and altered storage stability. pH values (5.1–5.6) and total ash content reflected seasonal differences in mineral absorption. Color parameters showed a notable decrease in L*, consistent with chlorophyll degradation over time. Mineral composition varied significantly, with potassium decreasing and calcium increasing toward the end of the season, while microelements such as manganese and iron were more abundant in early harvests. FTIR analyses revealed shifts in functional groups associated with polyphenols and other biochemical components. Aroma profiling showed that total volatile compounds peaked in July, whereas benzyl alcohol and benzaldehyde increased in later harvests. Antioxidant activity declined from 49.83 to 27.74 μmol TE/g, confirming that earlier harvests contain higher phenolic content and stronger antioxidant potential.

## Highlights

• Seasonal variation in tea quality was evaluated using physicochemical analyses combined with FTIR, mineral, and volatile profiling.
• Harvest-dependent molecular changes in polyphenols and minerals significantly influenced antioxidant activity and aroma composition.
• Molecular signatures were linked to bioactive and sensory properties, supporting harvest optimization and tea quality control.

## Graphical abstract

**Figure:**
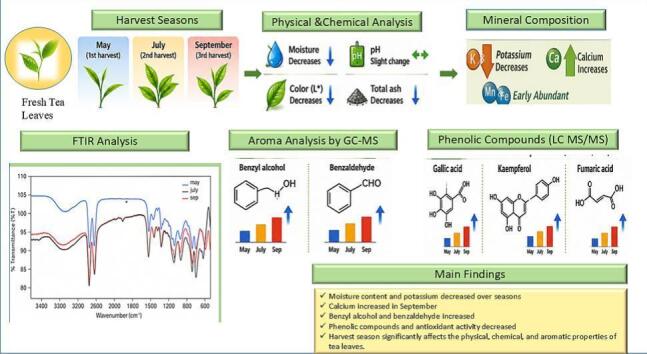

## 1 Introduction

Tea, predominantly derived from *Camellia sinensis*, is the second most consumed beverage globally after water, owing to its distinctive flavor, sensory appeal, and health-promoting properties. As a culturally and economically significant agricultural product, tea holds an important place in Turkey, particularly in the Eastern Black Sea region. This region, with its humid climate, consistent rainfall, and fertile volcanic soils, provides optimal growing conditions for tea cultivation. Turkish black tea is especially recognized for its rich color and flavor profile, which are significantly influenced by both environmental factors and seasonal variation. The unique dark hue and bright copper appearance of Turkish black tea are outcomes of fermentation, an essential step in black tea processing that enhances color and contributes to the development of characteristic aromatic compounds (Zeng et al., 2022).

Seasonality plays a crucial role in determining tea quality. Leaves harvested in early spring, particularly in May, are generally younger, more tender, and exhibit higher concentrations of bioactive compounds compared to those collected in later months such as July or September (Alasalvar et al., 2012). These spring-harvested leaves are richer in polyphenols, especially catechins like epicatechin (EC) and epigallocatechin (EGC), which are known for their antioxidant activities and contribute to the health benefits associated with tea consumption (Dai et al., 2015; Türkmen & Velioğlu, 2007). Additionally, other key compounds such as caffeine, flavonoids, and amino acids—particularly theanine—play essential roles in shaping the flavor, aroma, and functional properties of tea. Theanine, for instance, imparts a desirable umami taste, further enhancing consumer appeal (Yurteri et al., 2019).

The antioxidant capacity of black tea is closely linked to its polyphenolic composition, which evolves during fermentation. This process converts catechins into theaflavins and thearubigins, compounds that not only influence tea's sensory attributes but also enhance its antioxidant potential. Several studies have indicated that teas harvested in spring exhibit higher antioxidant levels than those collected in summer or autumn (Alasalvar et al., 2012; Balcı & Taban, 2018), suggesting a significant impact of harvesting time on tea quality. Moreover, the chemical and aromatic characteristics of Turkish tea are also shaped by post-harvest processing methods. High-grade teas produced by facilities such as ÇAYKUR are distinguished by their complex aroma profiles and strong sensory appeal, largely due to the presence of specific volatile organic compounds generated during processing (Alasalvar et al., 2012). In contrast, lower-grade teas often present diminished flavor and aroma, underscoring the importance of both harvest timing and processing technique in quality determination.

Furthermore, the biochemical composition of tea is influenced by environmental factors such as soil nutrient content and seasonal climatic variations. Cooler growing periods have been associated with increased nutrient uptake, particularly nitrogen and potassium, which contribute to improved flavor and enhanced bioactivity of the final product (Yang et al., 2022). The interaction between these agroecological conditions and the plant's physiology plays a vital role in defining tea's chemical makeup, particularly its phenolic and aromatic constituents (Kafkas et al., 2009). Given the substantial impact of seasonal and environmental factors on tea quality, this study aims to investigate how different harvesting periods affect the physical, chemical, and aromatic characteristics of Turkish tea.

The present study examines the impact of different harvest periods on the physical, chemical, and aromatic properties of tea. A comprehensive set of parameters — including color characteristics, moisture content, ash levels, mineral composition, phenolic content, antioxidant capacity, and aroma profiles — was evaluated to capture seasonal variability across the May, July, and September harvest periods. Phenolic composition and antioxidant capacity were further assessed to reveal the bioactive potential of the leaves, while a detailed aroma profile was generated to capture changes in volatile compounds between seasons.

Previous studies have widely examined the effect of seasonal variation on tea quality, but have mostly focused on isolated aspects such as chemical composition, antioxidant activity, or aroma profiling (Dai et al., 2015; Lee et al., 2013; Zeng et al., 2022). Unlike these (previous) studies, which typically consider a limited set of quality indicators or a single seasonal comparison, the present research systematically integrates physical properties, chemical and mineral composition, bioactive characteristics, and aroma profiles of tea samples collected from three distinct harvesting periods (May, July, and September) within the same production year and under comparable environmental and processing conditions. This integrative, single-year framework — combined with multivariate (PCA) interpretation of the aroma and phenolic data — enables a synchronized assessment of seasonal effects and a more robust interpretation of the relationships between compositional changes and sensory-related attributes than can be obtained from single-parameter studies.

## 2 Materials and methods

### 2.1 Sample collection and preparation

Fresh tea leaves (*Camellia sinensis*) were collected from a tea plantation located in the Eynesil district of Giresun Province, Türkiye, at an altitude of approximately 168 m above sea level, a region characterized by a humid climate and acidic soils (for Giresun pH values 3.84–4.66) typical of the Eastern Black Sea tea-growing areas (Turan et al., 2020). The tea plants were raised from seedlings originally sourced from the Of district of Trabzon province and were approximately 5 years old at the time of harvest. The tea cultivar was not formally identified; seedlings were locally sourced from the Of district of Trabzon, where *Camellia sinensis* var. *sinensis* is predominantly cultivated. Tea samples were collected on May 10, July 15, and September 20, 2025, corresponding to the first, second, and third harvest periods, respectively. These three periods correspond to the first, second, and third commercial harvest flushes practised in the Giresun–Eynesil district and together span the full commercial harvest window of the region; they therefore represent agronomically meaningful sampling points rather than arbitrary time intervals. Leaves were hand-plucked following standard regional harvesting practices (one bud with two leaves), and all samples were obtained from a single producer to minimise variability arising from cultivation and processing conditions.

Immediately after harvesting, the fresh leaves were transported to the laboratory in insulated containers to preserve their quality. The samples were washed with distilled water and air-dried under laboratory conditions. Subsequently, the samples were dried in a laboratory oven until constant weight was achieved, defined as a difference of less than 0.001 g between two consecutive measurements. The dried samples were then ground into a fine powder and stored in airtight containers until analysis.

For each harvest period, a single representative composite sample was prepared by pooling freshly plucked leaves collected from several tea bushes distributed across the same plantation, homogenised, and subsequently analysed in triplicate (technical replicates, *n* = 3).

### 2.2 Physicochemical analysis

Moisture content was determined by oven-drying 5 g of fresh leaves at 105 °C for 24 h, followed by cooling in a desiccator. Moisture (%) was calculated as the weight loss relative to the initial sample weight. PH was measured in a 10 g leaf homogenate prepared with 50 mL distilled water using a calibrated digital pH meter. Total ash content was determined by incinerating 2 g of dried leaves in a muffle furnace at 550 °C until a white ash formed. Total ash (%) was calculated based on the weight of the residue relative to the sample. Color of tea leaves were measured using a Hunter Lab colorimeter (Color Flex, Hunter Associates Laboratory Inc., Reston, Virginia, USA). The color values were expressed as L* = lightness; a* = red/green coordinate; b* = yellow/blue coordinate. All analyses were performed in triplicate, and the results are expressed as mean ± standard deviation.

### 2.3 Mineral composition analysis

Elemental composition of tea leaf samples was determined using an Energy Dispersive X-ray Fluorescence Spectrometer (Shimadzu EDX-7000, Kyoto, Japan). The instrument was equipped with a 50 kV Rh-anode X-ray tube, a high-resolution silicon drift detector (SDD), and an automatic five-position primary filter system. Prior to analysis, the spectrometer was allowed to thermally stabilize for 20–30 min and automatic energy calibration was completed using the built-in reference materials. Results are reported as mg element per g dry weight (or % *w*/w for major elements).

Measurement conditions were as follows: Tube voltage: 15–50 kV (automatically optimized), tube current: 10–1000 μA, collimator diameter: 10 mm, counting time: 60 s per measurement, detector: SDD with energy resolution <135 eV and atmosphere: air.

### 2.4 FTIR analyses

FTIR analyses were applied to investigate the alterations in the functional groups found in tea leaves during different harvest seasons. About 1–2 mg of tea powder were deposited between two disks of KBr, and then IR spectra were recorded using a FTIR spectrometer (FTIR 100, Perkin Elmer Incorporation, USA). The FTIR spectra of the samples were measured in the 4000–650 cm^−1^ region at room temperature.

### 2.5 Extraction of aroma compounds

Using the solid phase microextraction (SPME) method, aroma compounds were extracted. The fiber used in the extraction process has 50/30 μm thickness of polydimethylsiloxane/divinylbenzene/carboxen (PDMS/DVB/CAR). For analysis, 3 g of sample was weighed into 10 mL vials, sealed, and for thirty min, the SPME fiber was in contact with the vial's headspace. The temperature of the samples was adjusted to 40 °C, and the fiber adsorbing volatile compounds was injected into the GC (Kaftan & Elmaci, 2011). Analyses were applied in triplicate.

#### 2.5.1 Conditions for GC-FID and GC–MS

The evaluation of aroma compounds was realized using a GC equipped with a FID (Agilent 6890 N), while identification was performed using a mass spectrometer (MS) coupled to the GC (Agilent 5975B VL MSD). In this system, at the column outlet, the sample is divided equally using a special splitter (Dean switch-Agilent); the first part is directed to the FID and the second part is sent to the MS detector.

Using a DB-WAX capillary column of 60 m × 0.25 mm × 0.4 μm, aroma component separation was accomplished. The temperature of the injector was set at 220 °C and the detector at 250 °C. The column temperature began at 60 °C, then ramped at a rate of 2 °C/min to 220 °C, followed by a ramped at 3 °C/min to 245 °C, where it remained constant for 20 min. Three milliliters of sample were introduced into the GC. Helium was used as a carrier gas with 1.5 mL/min of flow rate. Temperatures of injection and detection part were applied at 250 °C. The injector type and temperature program for the MS used in aroma compound identification were identical to those of the GC. Helium flow rate was set to 1.5 mL/min. The ionization energy, ion source temperature and the quadrupole temperature of the MS were adjusted to 70 eV, 250 °C, and 120 °C, respectively. Scanning was performed at 1 s intervals over a mass/charge (*m*/*z*) range of 29˗350. By comparing the mass spectra of the injected reference compounds with the mass spectra of the compounds, the peak was identified and those stored in aroma compound libraries in the computer memory (Wiley 7.0, NIST-98, and Flavor.2 L) for compounds without standards. The amounts of volatile compounds were determined using the internal standard method after peak identification (Selli & Kelebek, 2011). Each analysis was conducted in triplicate.

#### 2.5.2 Calculation of aroma compound quantities

The amounts of aroma compounds by internal standard method were calculated using eq. 1, after calibration curves were obtained from standard compounds. Every compound's response factor was considered during the calculation.

(1) $$C_{i}=\left(A_{i}\;|\;A_{\mathrm{std}}\right)\times C_{\mathrm{std}}\times \mathrm{RF}\times \mathrm{HF}$$

*C*_*i*_: Aroma compound concentration,

*A*_*i*_: Aroma compound peak area,

*A*_*std*_: Internal standard peak area,

*C*_*std*_: Internal standard concentration (5 μL/100 mL),

*RF*: Response factor,

*HF*: Factor of calculation (factor used to convert the sample quantity to kilograms).

### 2.6 Determination of phenolic compounds

Five milliliters of methanol:water (60:40) were applied to 2.5 g of tea leaves. After two minutes of vortex mixing, the mixture was centrifuged for ten minutes at 3600 rpm. A membrane filter with a hole size of 0.45 μm was used to filter the collected methanolic extract after the procedure was repeated three times. ChemStation software running on Windows NT powered an Agilent 1100 HPLC machine (Agilent Technologies, Palo Alto, CA, USA). The HPLC apparatus was used in combination with a diode array detector (DAD). The column utilized was a Phenomenex Luna reversed-phase C-18 column (4.6 mm × 250 mm, 5 μm) (Torrance, CA) with a flow rate of 0.5 mL/min and an injection volume of 10 μL. The mobile phase consisted of two solvents: Solvent A, water/formic acid (99, 1; *v*/v) and Solvent B, acetonitrile/solvent A (60,40; v/v). Phenolic compounds were eluted under the following conditions: 0.5 mL/min flow rate and temperature set at 25 °C, DAD was set to 280, 320 and 360 nm for real-time monitoring of peak intensity. The identification and assignment of each compound was performed by comparing retention times and UV spectra with authentic standards and further confirmed by LC-MS/MS analysis.

### 2.7 Determination of antioxidant capacity (DPPH Method)

The antioxidant activity of green tea leaves was evaluated using the DPPH (2,2-diphenyl-1-picrylhydrazyl) radical scavenging test and expressed as μmol Trolox equivalents (TE) per gram of dry weight. Samples of dried and powdered tea leaves (0.1 g) were extracted using 10 mL of 80% methanol by sonication for 30 min and centrifugation at 10,000 ×*g* for 10 min. 100 μL of the sample extract was combined with 3.9 mL of a 0.1 mM DPPH solution made in methanol. The mixture was left in the dark at room temperature for 30 min, and absorbance was measured at 517 nm using a UV–Vis spectrophotometer. The results were calculated from the Trolox calibration curve (0–200 μM) and expressed as μmol TE/g dry weight. All measurements were repeated three times.

### 2.8 Statistical analysis

Statistical analyses were performed using the SPSS 22.0 software package (SPSS Inc., Chicago, IL, USA). Analysis of variance (ANOVA) was used to evaluate the experimental results. In order to determine statistically significant differences between the data sets, The Duncan multiple range test was applied. Differences were evaluated that there was a significant level at the *p* < 0.05. At the same time, principal component analysis (PCA) was applied to show the similarities/differences in aroma and phenol compounds in different harvest period samples in a biplot graphic. The PCA was conducted separately for aroma and phenolic compounds using a data matrix comprising the mean values of three harvest periods (May, July, September). The resulting matrices consisted of 3 observations × 44 aroma compounds and 3 observations × 19 phenolic compounds, respectively. As the number of observations was limited to three, only two principal components could be extracted (n − 1 = 2), and therefore the cumulative explained variance reaches 100% in both analyses, which reflects the mathematical constraints of the dataset rather than overfitting. Reported means ± standard deviations and the associated ANOVA and Duncan multiple-range comparisons are based on three technical replicates of a single composite sample per harvest period; they therefore characterise analytical reproducibility and the consistency of differences among the composite samples rather than between-collection biological variability.

## 3 Results and discussion

### 3.1 Influence of harvest timing on moisture, pH, Ash content and color of tea leaves

According to meteorological data obtained from the Turkish State Meteorological Service (Turkish State Meteorological Service [MGM], 2025) for the Giresun region in 2025 (Table 1), the average temperature rose from 15.9 °C in May to 25.1 °C and remained at a relatively high level in September (21.2 °C), while precipitation increased from 68.4 mm in May to 126.9 mm in September. The present study demonstrated significant variations in moisture, pH, total ash content and color properties of tea leaves across different harvest periods (Table 2). Moisture levels declined from 75.30% in May to 70.50% in September; this is consistent with reports indicating that leaf water content decreased in parallel with seasonal progression due to rising temperatures and increased transpiration rates (Langaroudi et al., 2023; Taiz et al., 2015).

**Table 1:** Average temperature and total precipitation values.

Harvest Period	Average Temperature (°C)	Total Precipitation (mm)
May	15.9	68.4
July	25.1	52.4
September	21.2	126.9

**Table 2:** Changes in moisture, pH, total ash content and color of fresh tea leaves collected from different harvest periods.

Physical Properties of Tea Leaves⁎	Harvest Period		
	May	July	September
Moisture (%)	75.30 ± 1.05^c^	72.40 ± 0.85^b^	70.50 ± 0.57^a^
pH	5.20 ± 0.15^a^	5.10 ± 0.23^a^	5.60 ± 0.15^b^
Total Ash Content (%)	4.98 ± 0.02^c^	4.83 ± 0.03^b^	4.63 ± 0.02^a^
Color Properties			
L⁎	73.24 ± 1.05^c^	65.33 ± 0.87^b^	58.45 ± 0.96^a^
a⁎	28.33 ± 0.77^c^	20.56 ± 1.63^b^	17.54 ± 0.86^a^
b⁎	13.21 ± 0.55^a^	17.85 ± 1.04^b^	20.23 ± 0.92^c^

⁎ Results are the mean value of three replication and standart deviation (mean ± std. dev.) of tea leaves. Different letters in the same row are statistically significant (*p* < 0.05).

Despite the increase in precipitation, high temperatures during the summer are likely to increase evapotranspiration and physiological water loss in tea leaves (Jones, 2014). Therefore, the decrease in moisture content observed during later harvest periods cannot be attributed solely to precipitation patterns; instead, it can be more reasonably explained by temperature-induced transpiration and increased metabolic activity. This suggests that temperature may play a more dominant role than precipitation in determining seasonal leaf moisture dynamics.

PH values ranged from 5.1 to 5.6, reflecting potential influences of soil properties and seasonal nutrient dynamics. Previous study emphasized that soil pH critically affects mineral uptake and overall tea quality (Jia et al., 2024), supporting the relevance of the observed fluctuations. Total ash content decreased from 4.98% in May to 4.63% in September, indicating seasonal variation in mineral accumulation. Similar trends have been reported, showing that mineral composition of tea shoots differs significantly with harvest season (Tang et al., 2020). In line with Erturk et al. (2010), such changes may also impact the antioxidant potential and nutritional value of tea. In summary, the findings are consistent with previous studies indicating that seasonal variations and environmental conditions exert a significant impact on the chemical composition of tea leaves. Continuous assessment of these factors is therefore crucial for optimizing tea quality and supporting sustainable cultivation practices.

The color measurements, assessed in May, July and September, reveal significant seasonal variations in L*, a*, and b* values (Table 2). The L* values, representing lightness, decreased markedly from 73.24 in May to 58.45 in September, indicating that leaves harvested later in the season were darker and less vivid. This decline suggests greater chlorophyll degradation during the warmer months, as higher temperatures are known to accelerate pigment breakdown (Huang et al., 2024). A reduction in lightness has also been linked to decreased freshness and altered flavor characteristics, since lighter leaves typically exhibit higher amino acid and polyphenol concentrations that contribute to a fresher taste profile (Ge et al., 2024). Similarly, the a* values dropped from 28.33 in May to 17.54 in September, reflecting a reduction in red pigmentation over successive harvests. This trend may be associated with declining anthocyanin content or shifts in phenolic compound composition, both of which influence the sensory attributes of tea (Maritim et al., 2021). Such changes could contribute to diminished brightness and complexity in the final infusion. Conversely, the b* values increased from 13.21 in May to 20.23 in September, demonstrating a notable rise in yellow pigmentation. This shift can be attributed to the progressive accumulation of carotenoids as chlorophyll content declines. Carotenoids play an essential protective role during stress conditions, particularly under high temperatures (Xu et al., 2024). Their higher levels in later harvests may also affect the oxidative and enzymatic pathways during tea processing, ultimately altering both the flavor and visual quality of the final product. The results emphasize the importance of harvest timing as a determinant of tea quality, linking biochemical pigment changes with both aesthetic and flavor outcomes.

2.1 Influence of Harvest Period on Mineral Profile of Fresh Tea Leaves

The mineral composition of tea leaves collected in three different harvest periods (May, July, and September) (Table 3) showed clear seasonal variations, indicating that nutrient accumulation in *Camellia sinensis* is strongly influenced by leaf age, physiological activity and environmental conditions. Overall, the first harvest (May) exhibited the highest concentrations for most macro- and microelements, while the third harvest (September) generally showed reduced levels, with a few notable exceptions such as Ca, Cr and Pb. Potassium (K), the most abundant element in all samples, presented a pronounced decrease from 51,080 mg/kg in May to 11,640 mg/kg in September. Similar decreasing trends were observed for phosphorus (P) and sulfur (S), both essential nutrients involved in energy transfer and amino acid metabolism. These patterns are consistent with the findings of Tang et al. (2020), who reported that spring tea leaves accumulate higher levels of N, P and K compared to later harvests, reflecting the greater metabolic activity of young leaves.

**Table 3:** Mineral composition of fresh tea leaves from different harvest periods.

Minerals	Concentration (mg/kg) ⁎		
	May	July	September
Potassium (K)	51,080.00 ± 30.41^c^	36,540.00 ± 35.59^b^	11,640.00 ± 3.06^a^
Calcium (Ca)	nd	3080.00 ± 29.44^a^	4270.00 ± 17.32^b^
Phosphorus (P)	5430.00 ± 17.32^c^	4600.00 ± 39.37^b^	980.00 ± 13.23^a^
Sulfur (S)	4290.00 ± 13.23^c^	3320.00 ± 24.83^b^	1580.00 ± 14.80^a^
Chlorine (Cl)	802.80 ± 8.66^c^	483.70 ± 14.14^a^	561.80 ± 10.44^b^
Manganese (Mn)	2690.00 ± 10.58^c^	930.00 ± 21.21^b^	650.00 ± 11.27^a^
Iron (Fe)	230.00 ± 6.08^c^	210.00 ± 5.52^b^	70.00 ± 1.14^a^
Zinc (Zn)	90.00 ± 4.36^b^	70.00 ± 7.07^a^	nd
Copper (Cu)	60.00 ± 3.46^b^	50.00 ± 4.80^a^	60.00 ± 1.01^b^
Rubidium (Rb)	80.00 ± 5.00^b^	50.00 ± 4.14^a^	nd
Cadmium (Cd)	nd	nd	nd
Lead (Pb)	nd	nd	2.60 ± 0.78
Mercury (Hg)	nd	nd	nd
Antimony (Sb)	nd	nd	nd
Chromium (Cr)	2.90 ± 0.12^b^	2.40 ± 0.10^a^	9.30 ± 0.18^c^
Bromine (Br)	1.20 ± 0.02^b^	1.20 ± 0.01^b^	0.70 ± 0.03^a^

Nd: not detected.

⁎ Results are the mean value of three replication and standart deviation (mean ± std. dev.) of tea leaves. Different letters in the same row are statistically significant (*p* < 0.05).

In contrast, calcium (Ca) exhibited an opposite trend: it was undetectable in the first harvest but increased significantly in July (3080 mg/kg) and September (4270 mg/kg). This pattern can be explained by the low mobility of Ca in plant tissues, leading to preferential accumulation in mature leaves. Similar observations have been reported by Liu et al. (2022), who found that immobile elements such as Ca and Mn tend to accumulate more in mature or post-pruning leaves of tea plants. From a plant-physiological perspective, these contrasting trends reflect differences in element mobility and leaf developmental stage. Potassium (K), phosphorus (P) and sulfur (S) are relatively mobile elements linked to active metabolic processes, so their higher concentrations in the early (May) harvest are consistent with the greater physiological activity of young, actively growing leaves. In contrast, calcium (Ca) is relatively immobile in plant tissues and is associated primarily with structural functions such as cell-wall stabilisation and thickening; its progressive accumulation across the later harvests is therefore consistent with leaf maturation and ongoing structural development in older tissues.

Among micronutrients, manganese (Mn), iron (Fe), zinc (Zn) and rubidium (Rb) showed their highest levels in May and progressively decreased across the season. Mn declined from 2690 to 650 mg/kg and Fe from 230 to 70 mg/kg between May and September. These results agree with previous observations from South India, where mineral concentrations such as K, P, Ca, Mn, Fe and Zn were shown to vary seasonally in tea leaves (Seenivasan et al., 2008). Copper (Cu), however, remained relatively stable across the three harvests, suggesting that its uptake and translocation are less sensitive to seasonal fluctuations.

Halogens also showed variable seasonal responses: chlorine (Cl) decreased from May to July but rose again in September, while bromine (Br) showed a gradual decline. Regarding heavy metals, cadmium (Cd), mercury (Hg) and antimony (Sb) were not detected in any harvest period, indicating minimal contamination risk. In contrast, lead (Pb) was detected only in the September harvest (2.6 mg/kg), suggesting either atmospheric deposition during late season or accumulation in older leaves. Previous studies have highlighted that seasonal and physiological factors influence heavy metal accumulation in tea plants. For instance, Peng et al. (2018) and Zaman et al. (2024) observed that lead (Pb) tends to accumulate more in mature leaves, while chromium (Cr) concentrations can increase during the later growth season, indicating a progressive accumulation of certain metals over time.

These mineral patterns coincide with broader metabolic changes driven by season. For instance, Liu et al. (2022) demonstrated that catechin biosynthesis and related gene expression differ significantly between spring and autumn flushes of tea, reflecting shifts in leaf metabolism across the growing season. The high concentrations of K, P, Mn and Fe in the first harvest may provide essential support for these biosynthetic activities, contributing to the generally higher biochemical quality of spring tea.

### 3.2 Influence of Harvest Period on FTIR Spectroscopy of Fresh Tea Leaves

**Fig. 1:**
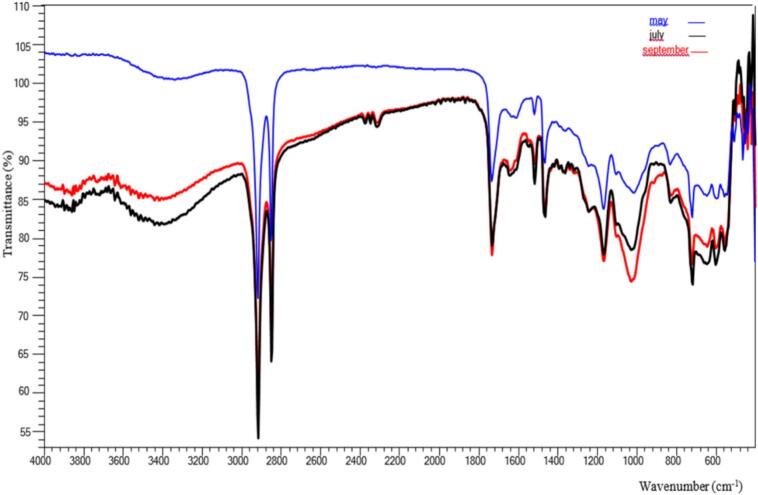
FTIR spectra of tea leaves harvested in May, July, and September.

Table 4 summarizes the main absorbance bands seen in the FTIR spectrum of tea leaves, the functional groups to which these bands correspond, and the compounds to which these groups are associated. FTIR spectra of tea leaves harvested in May, July, and September show characteristic vibrational bands associated with polyphenols, proteins, lipids, and carbohydrates (Fig. 1).

**Table 4:** FTIR peak assignments of tea leaves indicating corresponding functional groups and associated biochemical compounds.

	Functional Group	Assignment
3600–3200	O–H stretching (broad)	Polyphenols, cellulose, moisture, hydrogen bonding
∼2920	CH₂ asymmetric stretching	Lipids, aliphatic structures
∼2850	CH₂ symmetric stretching	Lipids, fatty acids
1740–1650	C=O stretching	Polyphenols, flavonoids, esters
1630–1600	C=C stretching	Aromatic rings, catechins, tannins
1540–1510	Aromatic skeletal vibration	Phenolic compounds
1450–1420	CH₂ bending	Lipid chains, lignin-related components
1370–1310	C–H bending	Cellulose, hemicellulose
1260–1200	C–O stretching / phenolics	Flavonoids, caffeine partial vibrations
1150–1030	C–O–C and C—O stretching	Cellulose, hemicellulose, sugars
1030–980	C–N and C—O stretching	Caffeine, amino acids
900–700	Aromatic C—H out-of-plane bending	Polyphenols, lignin-like structures

As seen in Fig. 1, while the overall spectral profiles are similar, seasonal differences are evident due to changes in peak intensities. The broad band at 3300–3400 cm^−1^ is attributed to the O—H stretching vibrations of phenolic hydroxyl groups and moisture, a characteristic feature observed in tea and plant materials (Liu et al., 2015). The relatively higher transmittance in the May sample suggests a decrease in phenolic compounds with lower moisture or hydroxyl content in early-season leaves. Strong absorbances at 2920 and 2850 cm^−1^, corresponding to asymmetric and symmetric C—H stretching of aliphatic –CH₂ groups, are associated with lipid structures (Brza et al., 2020) These peaks are more pronounced in the July and September samples, indicating an increase in lipid-related components as the season progresses. The region around 1600–1650 cm^−1^ represents the aromatic C=C stretching of amide I of polyphenols and/or proteins, as reported by Giorgini et al. (2023) Similarly, the 1500–1550 cm^−1^ band corresponds to the amide II vibrations of proteins, reflecting N—H bending and C—N stretching in protein structures (Giorgini et al., 2023).

The differences in these regions between the three harvest periods likely reflect seasonal changes in both polyphenolic composition and protein content. The 1000–1200 cm^−1^ fingerprint region, dominated by the C—O and C–O–C stretching of carbohydrates, is a well-established indicator of polysaccharide structures in plant tissues (Giorgini et al., 2023; Jóźwiak et al., 2021). The July sample exhibits relatively stronger absorption in this region, indicating increased carbohydrate accumulation mid-season.

FTIR spectroscopy has been widely used to distinguish tea samples according to harvest time, geographical origin, and biochemical composition (Yorulmaz et al., 2025). The trend observed in our data (gradual changes in O—H, C—H, and C—O bands) is consistent with findings that tea leaf metabolites and phenolic composition vary with seasons (Giorgini et al., 2023; Yorulmaz et al., 2025).

### 3.3 Influence of harvest period on aroma compounds of fresh tea leaves

Table 5 shows the aroma compounds of fresh tea leaves harvested in May, July, and September. According to the data obtained, total aroma compounds were 2817 μg/kg, 10,019 μg/kg, and 8468 μg/kg in May, July, and September, respectively. The highest levels of aroma compounds were observed in July, followed by a slight decrease in September.

**Table 5:** Aroma compounds of fresh tea leaves from different harvest periods.

No	Aroma Compounds	RT	Concentration (μg/kg)*		
			May	July	September
1	3-Hydroxy butanal	11.21	91.48 ± 3.25^a^	427.43 ± 30.51^c^	176.09 ± 10.75^b^
2	Hexanal	13.29	205.23 ± 18.42^a^	468.85 ± 16.17^b^	456.02 ± 11.54^b^
3	(*E*)-3-Penten-2-one	15.08	nd	170.15 ± 6.72	nd
4	(*E*)-2-Pentenal	15.25	nd	77.67 ± 5.23	nd
5	(*Z*)-2-Pentenol	16.85	nd	209.95 ± 1.06	nd
6	2-(2-Propenyl) furan	19.19	nd	44.11 ± 3.66	nd
7	(*E*)-2-Hexenal	19.74	84.72 ± 1.99^a^	528.02 ± 18.33^c^	280.69 ± 16.41^b^
8	2-Pentyl furan	20.76	nd	88.41 ± 4.60^b^	71.55 ± 1.41^a^
9	Ethyl hexanoat	20.94	nd	nd	85.44 ± 6.72
10	Octanal	23.53	47.91 ± 4.52^a^	108.26 ± 4.22^c^	76.97 ± 5.47^b^
11	1-Octyn-3-ol	23.98	34.59 ± 1.50^a^	125.14 ± 2.57^c^	104.73 ± 4.12^b^
12	(*Z*)-2-Heptenal	24.94	177.78 ± 12.80^a^	551.06 ± 24.51^c^	342.60 ± 18.29^b^
13	6-Methyl-5-hepten-2-one	25.54	nd	62.60 ± 2.12	nd
14	Hexanol	26.19	42.65 ± 3.85^a^	75.07 ± 3.07^b^	99.10 ± 4.68^c^
15	(*E*)-4-Hexenol	27.40	34.58 ± 1.08^a^	90.66 ± 5.19^b^	110.76 ± 5.91^c^
16	(*E,E*)-2,4-Hexadienal	28.08	62.75 ± 4.83^a^	491.33 ± 18.82^c^	125.95 ± 9.08^b^
17	Nonanal	28.25	238.39 ± 14.73^a^	655.51 ± 20.28^c^	410.95 ± 13.21^b^
18	(*E*)-3-Octen-2-one	28.62	41.60 ± 2.90^a^	103.62 ± 6.07^c^	82.38 ± 1.95^b^
19	(*E*)-2-Octenal	29.54	84.88 ± 1.97^a^	251.96 ± 8.84^c^	201.72 ± 7.24^b^
20	Cis-Linalool oxide (furanoid)	30.14	56.49 ± 4.81^a^	73.51 ± 2.60^b^	91.48 ± 3.45^c^
21	1-Octen-3-ol	30.20	89.57 ± 2.53^a^	270.22 ± 18.37^c^	150.89 ± 6.23^b^
22	(*E,E*)-2,4-Heptadienal	30.66	216.47 ± 18.25^a^	641.16 ± 17.94^b^	230.96 ± 12.77^a^
23	trans-Linalool oxide (furanoid)	31.25	221.02 ± 17.53^b^	251.98 ± 9.31^c^	197.94 ± 7.80^a^
24	Decanal	32.62	nd	167.11 ± 11.21^b^	93.40 ± 2.58^a^
25	Benzaldehyde	32.94	198.49 ± 6.51^a^	514.42 ± 29.03^b^	1446.85 ± 81.18^c^
26	Ethinamate	33.03	58.30 ± 1.00^a^	273.41 ± 8.11^b^	nd
27	(*E*)-2-Nonenal	34.09	nd	97.56 ± 7.11^b^	60.69 ± 3.15^a^
28	Linalool	34.33	62.81 ± 5.51^a^	386.53 ± 13.48^c^	196.57 ± 9.63^b^
29	Octyl chloroformate	34.90	nd	73.30 ± 5.78	nd
30	(*E,E*)-3,5-Octadien-2-one	35.41	nd	34.60 ± 2.68	nd
31	3-Decyn-2-ol	36.24	nd	37.21 ± 2.74	nd
32	2,5-Dimethylcyclohexanol	36.95	nd	91.48 ± 3.57	nd
33	(Z)-2-Octenol	37.31	nd	33.54 ± 1.49	nd
34	.alpha.-[(methylamino)methyl]-benzenemethanol	38.67	nd	34.61 ± 1.42	nd
35	(*E*)-2-Decenal	38.88	37.73 ± 2.06^a^	201.52 ± 18.03^c^	109.04 ± 8.24^b^
36	(3*R*,6*S*)-2,2,6-Trimethyl-6-vinyltetrahydro-2H-pyran-3-ol	42.75	78.12 ± 6.78^c^	44.62 ± 3.32^a^	66.20 ± 3.18^b^
37	Methyl salicylate	43.24	52.93 ± 3.60^a^	214.65 ± 9.15^b^	720.36 ± 16.59^c^
38	Quinuclidine	45.22	nd	62.53 ± 1.02	nd
39	Geraniol	45.36	68.95 ± 6.03^a^	171.20 ± 10.38^c^	76.44 ± 2.19^b^
40	Benzyl alcohol	45.96	374.63 ± 21.53^a^	1007.10 ± 34.73^b^	1918.85 ± 99.34^c^
41	(*E*)-1,10-Dimethyl-trans-9-decalinol	46.84	46.70 ± 3.64^a^	108.73 ± 6.39^b^	nd
42	Phenylethyl alcohol	47.05	108.32 ± 7.71^a^	529.92 ± 28.63^c^	483.44 ± 10.50^b^
43	trans-Beta.ionone	48.43	nd	81.61 ± 3.50	nd
44	Imidazole, 2-amino-5-[(2-carboxy)vinyl]	58.00	nd	86.19 ± 4.76	nd
**TOTAL**			**2817.11**	**10,018.51**	**8468.03**

* Results are the mean value of three replication and standart deviation (mean ± std. dev.) of aroma compounds of tea leaves collected at different harvest periods. ^a-c^Different letters in the same row are statistically significant (*p* < 0.05).

Furthermore, the number of compounds was determined to be 26 for May, 43 for July, and 28 for September. It is observed that July was the richest in aroma compounds, both in terms of diversity and quantity. Among the 44 identified volatiles, benzyl alcohol, nonanal, and benzaldehyde were distinguished as major contributors to the aroma profile due to their high concentrations and sensory relevance.

Benzyl alcohol concentrations increased significantly across harvest periods: 374.63 μg/kg in May 1007.10 μg/kg in July, and 1918.85 μg/kg in September. This trend reflects an approximately fivefold increase from early harvest to late harvest. Benzyl alcohol is widely accepted to contribute to the floral and sweet aroma notes in tea, and these notes originate primarily from phenylalanine-based metabolic pathways and glycosidic-linked precursors (Parveen et al., 2023; Zhang et al., 2022). The observed late-season peak suggests that leaf ripening and seasonal cues (e.g., decreasing daylight, cooler nights) increase the hydrolysis of glycosidic aroma precursors, thereby releasing higher amounts of benzyl alcohol (Parveen et al., 2023). Nonanal, (fatty, citrus wax) aldehyde typically derived from linoleic acid oxidation via LOX, reached the highest level (655.51 μg/kg) in July, followed by 238.39 μg/kg in May and 410.95 μg/kg in September.

The mid-summer peak for nonanal is consistent with previous findings that periods of high temperature increase lipid oxidation and aldehyde formation in tea leaves (Zheng et al., 2022). This suggests that enzymatic oxidation of unsaturated fatty acids in July-harvested leaves increased, producing higher nonanal content and contributing to the fresh/green citrus aroma notes. Benzaldehyde showed the most striking seasonal change, increasing from 198.49 μg/kg in May to 514.42 μg/kg in July and reaching 1446.85 μg/kg in September. Benzaldehyde is a major almond/hazelnut-like aromatic compound derived from phenylalanine metabolism or glycosidic precursor hydrolysis (Zheng et al., 2022). The significant increase in September suggests that the phenylpropanoid/benzenoid metabolic flux is strongly activated in late-season leaves, likely resulting from maturity and/or environmental stress responses (Zhang et al., 2022). When other compounds were examined, hexanal increased from ∼205 μg/kg in May to ∼469 μg/kg in July and remained high at ∼456 μg/kg in September. This is an aldehyde compound with green, grassy odors formed through lipid oxidation and enzymatic activity Lee et al., 2013; Grebenteuch et al., 2021). Linalool, a floral and sweet alcohol, showed a significant increase in July (∼386 μg/kg) compared to May (∼63 μg/kg), followed by a decrease in September (∼197 μg/kg). This suggests that floral volatiles accumulate midseason and are regulated by leaf maturation and environmental conditions (Lehto et al., 2003). In particular, methyl salicylate showed a dramatic increase (∼720 μg/kg) in September, from ∼53 μg/kg in May and ∼ 215 μg/kg in July. A previous study suggested that this likely reflects changes in leaf metabolism, ester formation, or selection of more mature leaves (Ma et al., 2019). In general, aldehydes such as (E)-2-hexenal and (E,E)-2,4-hexadienal reached maximum levels in July.

### 3.4 PCA Interpretation of aroma compounds across harvest periods

**Fig. 2:**
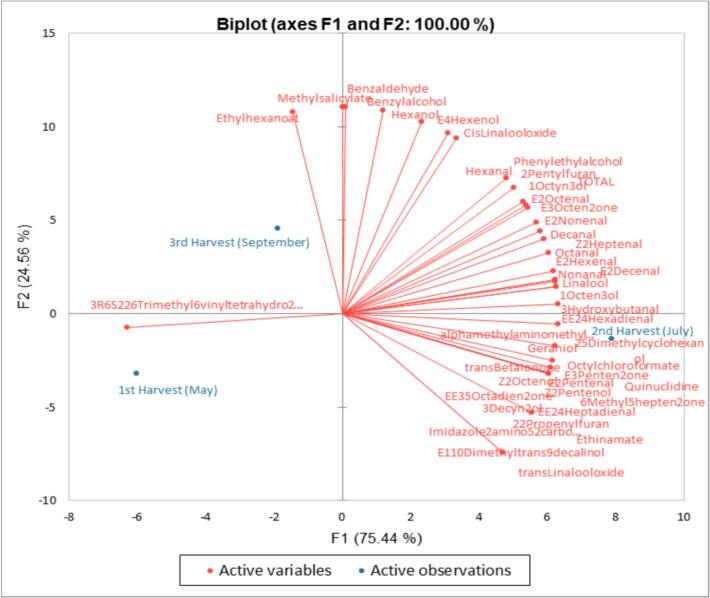
PCA biplot showing the distribution of aroma compounds in different harvest period samples.

The principal component analysis (PCA) successfully discriminated the samples obtained from the three harvest periods (May, July, and September) based on their volatile compound profiles (Fig. 2). The first two principal components explained 100% of the total variance, with PC1 accounting for 75.44% and PC2 for 24.56%, indicating a strong ability of the model to represent the overall variability within the dataset. The samples from the first harvest (May) were positioned on the negative side of PC1 and slightly negative along PC2, forming a distinct cluster separated from the other harvest periods. This separation is primarily associated with the high contribution of 3R-6S-2,2,6-trimethyl-6-vinyltetrahydro-2H-pyran-3-ol, which exerted a strong loading in the same direction. The dominance of this volatile suggests that the early harvest material possessed a more limited and specific aromatic profile, characterized by green and fresh sensory attributes.

In contrast, the second harvest (July) was located on the positive side of PC1, where the majority of volatile compound vectors were oriented. This indicates that the July samples exhibited the highest chemical diversity and intensity of aroma-active compounds. The predominance of these compounds corresponds to more fruity, floral, and citrus-like aromatic characteristics, suggesting that the mid-season harvest represents the aromatic peak of the plant material.

The third harvest (September) formed a cluster positioned on the negative side of PC1 but clearly positive along PC2, distinguishing it from the May samples. These volatiles are typically linked to sweet, floral, almond-like, and mildly fruity notes, suggesting that late-season material develops a more mature aromatic profile.

### 3.5 Influence of harvest period on phenol compounds and antioxidant activity of fresh tea leaves

Table 6 shows the concentration of phenolic compounds and antioxidant activities in tea leaves collected in three harvest periods (1st harvest: May; 2nd harvest: July; 3rd harvest: September). Two main phenomena emerge from the results: (1) a general decrease in many flavonoids and hydroxycinnamic-type compounds from early to late harvest and (2) selective increases or relative enrichments of certain phenolics (notably gallic acid and some stress-related compounds) in later harvests. These findings are consistent with numerous previous studies showing that harvest time/maturity strongly influences phenolic profiles in olives and other plant matrices (Deng et al., 2017; Zheng et al., 2022). According to the results, an interesting trend is observed in gallic acid, which was determined to have the highest amount in all harvest periods, with its concentration increasing from 64,284.19 μg/kg in May to 102,956.65 μg/kg in September. Increased gallic acid in later stages may reflect the gradual hydrolytic or oxidative degradation of larger polyphenols/tannins (e.g., gallotannins, ellagitannins) and subsequent accumulation of free gallic acid (Fernández-Poyatos et al., 2021). One study indicated that gallic acid acts as a defense mechanism against environmental stresses, such as increased fungal activity and UV stress in late summer, and may highlight an adaptation strategy that increases plant resistance during this period (Panzella et al., 2020). Similar findings were reported by Yao et al. (2005) who linked seasonal changes in the synthesis of phenolic compounds to environmental conditions, such as sunlight exposure, that affect metabolism. The concentration of fumaric acid decreased markedly from 47,844.36 μg/kg in May to 25,853.94 μg/kg by September. This decline can be linked to the plant transitioning from a growth phase to a reproductive stage, wherein metabolic pathways prioritize other compounds for defense and reproductive functions (Mohale et al., 2020).

**Table 6:** Phenolic compounds and antioxidant activities of fresh tea leaves collected at different harvest periods.

Phenolic Compounds	Concentration (μg/kg)*		
	May	July	September
Vanillic Acid	12,562.69 ± 13.08^c^	11,325.52 ± 19.31^b^	10,296.23 ± 15.72^a^
Acetohydroxamic Acid	3049.26 ± 8.72^c^	2880.93 ± 10.58^b^	2475.6 ± 7.81^a^
Syringic Acid	8847.73 ± 10.58^c^	8013.71 ± 13.08^a^	8299.69 ± 14.80^b^
Resveratrol	7681.81 ± 13.89^a^	10,571.59 ± 12.29^c^	10,544.49 ± 12.17^b^
Kaempferol	31,458.21 ± 8.72^c^	22,679.25 ± 15.72^b^	18,190.16 ± 14.80^a^
Ellagic Acid	15,714.95 ± 24.58^a^	20,953.27 ± 17.44^c^	18,549.76 ± 17.78^b^
Chlorogenic Acid	10,772.19 ± 15.87^c^	10,330.04 ± 12.17^b^	6037.19 ± 12.49^a^
Fumaric Acid	47,844.36 ± 27.78^c^	33,494.57 ± 23.81^b^	25,853.94 ± 20.95^a^
Gallic Acid	64,284.19 ± 38.97^a^	70,372.59 ± 34.70^b^	102,956.65 ± 38.22^c^
Protocatechuic Acid	10,234.65 ± 15.62^c^	9537.26 ± 13.89^b^	7661.93 ± 14.18^a^
Caffeic Acid	1658.87 ± 8.89^c^	1598.85 ± 8.19^b^	1580.30 ± 7.00^a^
Phloridzin dihydrate	2535.06 ± 16.64^ab^	2511.23 ± 13.89^a^	2561.81 ± 14.00^b^
Myricetin	6915.95 ± 9.64^c^	6188.54 ± 9.54^b^	5179.95 ± 11.36^a^
Hydoxycinamic Acid	37,733.16 ± 22.74^c^	27,718.80 ± 19.08^b^	22,717.62 ± 21.79^a^
Naringenin	1772.19 ± 11.27^c^	1221.26 ± 8.72^b^	1106.47 ± 10.82^a^
Quercetin	8311.48 ± 19.70^c^	5148.10 ± 16.46^b^	4507.89 ± 13.00^a^
Luteolin	5569.29 ± 17.58^c^	3444.11 ± 12.17^b^	2834.64 ± 13.23^a^
Salicylic Acid	19,651.59 ± 23.30^c^	18,414.90 ± 19.47^b^	17,776.98 ± 18.03^a^
4-Hydroxybenzoic Acid	3243.44 ± 10.44^c^	3142.05 ± 8.89^b^	2210.15 ± 10.44^a^
Antioxidant Activity (μmol TE/g)*	49.83 ± 0.18^c^	43.25 ± 0.20^b^	27.74 ± 0.15^a^

* Results are the mean value of three replication and standart deviation (mean ± std. dev.) of phenolic compounds and antioxidant activity of tea leaves collected at different harvest periods. ^a-c^Different letters in the same row are statistically significant (*p* < 0.05).

Hydroxycinnamic acid concentration is higher in the first harvest (May) and gradually decrease in the middle and late harvests. This supports the interpretation that young or actively growing tissues increase the biosynthesis of flavonoids and hydroxycinnamates, which are involved in UV protection, photoprotection, and antioxidant defense during periods of intense photosynthetic activity (Reungdech et al., 2019).

The concentration of vanillic acid was highest in May (12,562.69 μg/kg) and decreased in subsequent harvests, reaching 10,296.23 μg/kg in September. This decrease suggests that phenolic acids may be synthesized more actively during the early spring growth phase and that this may be a protective response against environmental stressors such as UV radiation and pathogens (Shahidi & Ambigaipalan, 2015). Xu et al. (2012) explain this decrease by decreasing secondary metabolite production as the growth cycle progresses or by changing the plant's need for these compounds. Notably, kaempferol showed a significant decline from 31,458.21 μg/kg in May to 18,190.16 μg/kg by September. Kaempferol is known for its antioxidant properties and its role in protecting plants against oxidative stress. The reduced levels in later harvests may indicate diminished stress responses or lower metabolic activity in the plants as they progress through the growing season (Ahmed et al., 2019; Xu et al., 2012). Similar to gallic acid, ellagic acid levels increased from 15,714.95 μg/kg in May to 20,953.27 μg/kg in July, before slightly decreasing to 18,549.76 μg/kg by September. This pattern suggests a peak in biosynthesis during the mid-growing season, likely reflecting optimal conditions for metabolite production (Ahmed et al., 2019; Xu et al., 2012). Interestingly, resveratrol content increased in the 2nd harvest (July) to 10,571.59 μg/kg and remained relatively stable in the 3rd harvest (10,544.49 μg/kg). Resveratrol is associated with a variety of health benefits, including anti-inflammatory properties, and its higher concentration suggests an ongoing response to stress factors throughout the growing season (Ghimire et al., 2021). The findings align with previous studies indicating that environmental stressors, particularly UV light and temperature changes, can significantly affect the biosynthesis of phenolic compounds in plants (Shahidi & Ambigaipalan, 2015). Phenolic compounds, including flavonoids and phenolic acids, contribute to the antioxidant capacity, sensory characteristics, and health benefits of tea (Mohale et al., 2020). The levels of phenolic compounds are directly related to the antioxidant activity and sensory qualities of tea. Higher concentrations in the initial harvest correspond with better health benefits, emphasizing the significance of the harvest period in quality assessment (Thongbai et al., 2025). Dose-response relationships between specific phenolics and health outcomes reinforce the importance of selecting optimal harvest times, as indicated by Jamir and Ajungla (2020), who noted variations in antioxidant capacities across different seasons (Ye et al., 2022). Furthermore, sensory characteristics, such as astringency and bitterness associated with phenolic compounds, are critically influenced by their concentration. Correlations between the amounts of flavanols and the perceived quality of tea illustrate the importance of timing in the harvesting process as described by Scharbert et al. (2004). Antioxidant activity values  of tea leaves, determined by the DPPH method, are shown in Table 5. The antioxidant activity of tea leaves harvested in May was 49.83 ± 0.18 μmol TE/g; in July, 43.25 ± 0.20 μmol TE/g; and in September, 27.74 ± 0.15 μmol TE/g. These values  demonstrate the effect of tea harvest time on antioxidant capacity. Previous studies have shown that antioxidant activity is affected by factors such as harvest time, environmental conditions, and tea processing. For example, Granato et al. (2014) reported that green tea generally had the highest antioxidant activity in DPPH tests conducted on various tea types (green, black, and white). The high antioxidant activity in May represents the period when the tea leaves are young and fresh. This is due to the higher polyphenol content of young leaves. However, senescent leaves harvested in July and September exhibit lower DPPH scavenging activity. In a previous study, Misra et al. (2008) noted that the antioxidant potential of young tea leaves was significantly superior to that of older leaves. The lower antioxidant activity of tea leaves harvested in September may have been influenced by both the decrease in polyphenol content and environmental factors (e.g., temperature and humidity). This suggests that biochemical changes in senescent leaves lead to a decrease in antioxidant components.

### 3.6 PCA interpretation of phenolic profiles across harvest periods

**Fig. 3:**
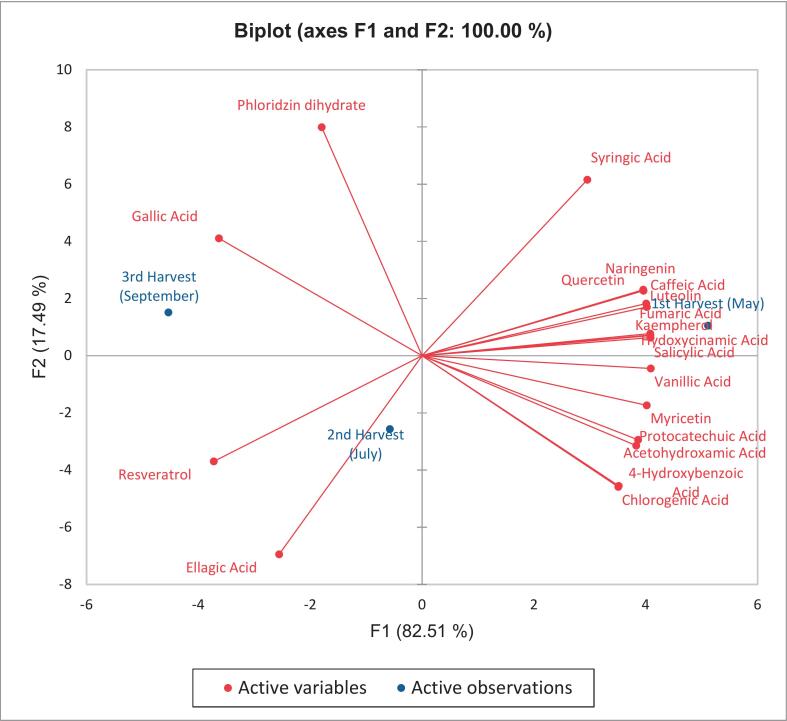
PCA biplot showing the distribution of phenol compounds in different harvest period samples.

The principal component analysis (PCA) biplot explains 100% of the total variance within the dataset, with F1 accounting for 82.51% and F2 for 17.49% of the total variation (Fig. 3). This indicates a highly effective dimensional reduction and a strong representation of the original variables in the first two components. The samples collected from different harvest periods (May, July, and September) were clearly separated along the two principal components, reflecting substantial differences in their phenolic compositions. 1st Harvest (May) samples are positioned on the positive side of the F1 axis, showing a strong correlation with quercetin, luteolin, kaempferol, caffeic acid, hydroxycinnamic acid, and salicylic acid. These compounds are predominantly flavonoids and hydroxycinnamic acids, which are known to accumulate during early developmental stages under high photosynthetic activity and UV exposure (Reungdech et al., 2019).

Therefore, the May harvest represents the period with the highest phenolic diversity and concentration, suggesting strong antioxidant potential. 2nd Harvest (July) samples appear in the negative quadrant of F1 and F2, closely associated with resveratrol and ellagic acid. These polyphenols are typically synthesized as part of stress-induced defense mechanisms, particularly under heat or oxidative stress (Rao & Zheng, 2025). The July period likely reflects a shift from biosynthetic flavonoids toward protective phenolics, which corresponds to environmental stress during mid-summer. 3rd Harvest (September) samples are located in the negative region of F1 and the positive region of F2, correlated mainly with gallic acid and phloridzin dihydrate. Both compounds are often related to oxidative polymerization processes and ripening-associated metabolism, indicating increased degradation or transformation of complex phenolics during fruit maturation (Fernández-Poyatos et al., 2021).

Although this study provides meaningful insights into seasonal variability in tea quality, some limitations should be noted. The analyses were based on a single composite sample per harvest period from one producer, which was intentionally designed to isolate the seasonal effect by minimising variability arising from cultivation and processing differences. Nevertheless, incorporating independent biological replicates across multiple producers and growing regions in future studies would further strengthen the generalisability of the findings.

It should be noted that the statistical differences observed in this study reflect analytical reproducibility among composite samples rather than independent biological variation. Therefore, the findings should be considered preliminary and should not be generalized to all tea plantations without further biological replication.

## 4 Conclusion

In conclusion, the findings of this study indicate that the highest moisture content was recorded in May, with significant decreases observed in subsequent harvests. This is indicative of physiological changes that occur as the leaves mature and are exposed to environmental stressors. The study also elucidated the relationship between harvest time and the mineral profile of tea leaves, revealing a trend toward decreasing macronutrient concentrations over time, while calcium content increased in subsequent harvests. This finding supports the notion that younger leaves accumulate higher levels of essential minerals, associated with higher antioxidant and phenolic profiles. Furthermore, principal component analysis (PCA) conducted on aroma compounds and phenolic profiles revealed clear clusters between harvest periods, reinforcing the idea that timing is crucial for achieving optimal flavor and aroma characteristics. The highest antioxidant activity values  were observed in the earliest harvest in May. In light of these results, it is clear that strategic planning in tea harvest timing will significantly impact the physical and chemical properties of the final product. The findings support further research examining the interactions of environmental conditions, leaf maturity and processing methods to develop best practices in tea cultivation that meet quality standards and consumer preferences.

## Author contribution

Hicran Uzun Karka: Methodology, formal analysis and investigation, writing-original draft preparation, writing-review and editing. Songul Kesen: Conceptualization, Methodology, formal analysis and investigation, writing-original draft preparation, writing-review and editing, supervision.

## CRediT authorship contribution statement

**Hicran Uzun Karka:** Writing – review & editing, Writing – original draft, Methodology, Investigation, Formal analysis. **Songul Kesen:** Writing – review & editing, Writing – original draft, Supervision, Methodology, Investigation, Formal analysis, Data curation.

## Funding

The authors thank the Scientific Research Projects (BAP) Coordination Unit of 10.13039/501100008836Gaziantep University for funding this research (Project Number: NTMYO.HZP.24.06).

## Declaration of competing interest

The authors declare that they have no known competing financial interests or personal relationships that could have appeared to influence the work reported in this paper.

## Data Availability

https://figshare.com/s/0815d92ed1038d96219e
